# Complete mitochondrial genome of *Lasiommata deidamia* and its phylogenetic implication to subfamily Satyrinae (Lepidoptera: Nymphalidae)

**DOI:** 10.1080/23802359.2021.1955029

**Published:** 2021-09-15

**Authors:** Yuxuan Sun, Chen Chen, Xuexia Geng, Jun Li

**Affiliations:** College of Life Science, Huaibei Normal University, Huaibei, P.R. China

**Keywords:** Nymphalidae, Satyrinae, *Lasiommata deidamia*, mitogenome, phylogeny

## Abstract

*Lasiommata deidamia* Eversmann taxonomically belongs to lepidopteran family Nymphalidae Rafinesque, 1815. The Complete mitochondrial genome (mitogenome) of the insect had been sequenced, with 15,244 bp of total length that has 81.12% AT content and contains a typical set of genes (13 protein-coding genes (PCGs), 22 tRNA genes and 2 rRNA genes) and a 417 bp AT-rich region. Another, 11 intergenic spacers (139 bp in total) and 16 overlaps (175 bp in total) have been founded. The longest interval is located between *trn^Gln^* and *nad2* while the maximum overlap is between *trn^His^* and *nad4*. All PCG genes are started with the ATN codons and stop at TAA codons except *cox1* which uses CGA as the initiation codon. No tandem repeat has been found in the AT-rich region. The phylogenetic tree inferred with Bayesian Inference based on all the 13 protein sequences of 45 mitogeomes reveals the phylogenetic relationships of the taxa in the subfamily Styrinae is (((Satyrini + Ypthimini) + (Amathusiini + Elymniini)) + Melanitini) and that within tribe Satyrini is ((((Lethina + Parargina) + Mycalesina) + Coenonymphina) +(Satyrina + (Melanargiina + Maniolina))).

For the advantages of small size (average 14–16 kb), conservative gene (13 proteins coding genes, 22 transfer RNA genes and 2 ribosome RNA genes), highly genic mutation in nature populations, maternal inheritance, nearly neutral evolution and easy to amplify, mitogenomes have been widely used for the researches on molecular systematics, population genetics, molecular evolution, comparative genomics and evolutionary genomics (Cameron 2014). The species of family Nymphalidae have been distinguished with other butterflies by the unique ventromesial surface of antennae with three longitudinal carinae separating two continuous sulci or shallow depressions on each segment (Ackery et al. 1999). Family Nymphalidae also has been proved monophyletic but its subdivision of has some uncertainty (Ackery et al. 1999). And new species, even new genera, have been discovered or revised (Freitas et al. 2010, 2011; Zacca et al. 2013; Nakahara et al. 2014; Barbosa et al. 2020). Thus, more mitogenome data will be helpful to make the subdivision of family Nymphalidae clearer.

The specimens of *L. deidamia* were netted from Xiangshan Mountain, Huaibei City, Anhui Province, China (116°48′34″ E, 33°59′1″ N). The total DNA was extracted according to the instruction book of DNeasy® Blood & Tissue Kit (QIAGEN, Germany) and its quantity and quality was evaluated by NanoDrop 2000c spectrophotometer (Thermo, USA) and 1% agarose gel electrophoresis. Primers to amplify mitochondrial DNA were designed according to the conservative regions in the published mitogenomes of lepidopteran insects and overlapping fragments were amplified using PrimeSTAR® GXL DNA Polymerase (Takara, China) according the instructions. Primers are listed in Supplementary Table 1. Amplified PCR productions were sequenced with Sanger dideoxy sequencing method by Shanghai Sequencing Department of Beijing Genomics institution (BGI). Complete mitochondrial DNA sequence was assembled using Lasergene DNASTAR kits, annotated using MITOS (Bernt et al. 2013) and manually verified with NCBI BLAST. Tandem repeats in AT-rich region were discovered with Tandem Repeats Finder Program (Benson 1999).

The complete mitogenome of *L. deidamia*s (GenBank accession number MG880214) is 15,244 bp in total with 81.12% AT content. The number and arrangement of the mitogenome genes just like most of lepidopteran mitogenomes with *trn^Met^*−*trn^Ile^*−*trn^Gln^* arrangement in which 23 genes (9 PCGs and 14 tRNAs) are located in the majority strand (J-strand) and 14 genes (4 PCGs, 8 tRNAs and 2 rRNAs) in the minority strand (N-strand). In this mitogenome, 11 intergenic spacers (139 bp in total) and 16 overlaps (175 bp in total) have been founded. The intergenic nucleotides vary from 2 to 51 bp and the longest interval locates between gene *trn^Gln^* and gene *nad2* while the overlap nucleotides vary from 1 to 62 bp and the maximum overlap lies between gene *trn^His^* and gene *nad4*. All PCGs stop at the terminated codon TAA and start with the initiative codons as ATN except for cox1 using CGA as initiation codon. The mitogenome also has a 417 bp AT-rich region containing an 'ATAGA + polyT' motif and no tandem repeat has been found.

Forty-five verified complete mitogenomes of the subfamily Satyrinae Boisduval, 1833 have been deposited in GenBank at present and *Polyura nepenthes* (Nymphalidae: Charaxinae) and *Calinaga davidis* (Nymphalidae: Calinaginae) were selected as out-group species. We downloaded all 13 protein sequences in these mitogenomes and alignment them using MAFFT software (Yamada et al. 2016). All aligned protein sequences were incorporated together to reconstruct phylogenetic tree using Bayesian Inference (BI) method with the amino acid substitution model mtREV24 + G + I+F . The result reveals the phylogenetic relationships of the taxa in the subfamily Satyrinae is (((Satyrini + Ypthimini) + (Amathusiini + Elymniini)) + Melanitini) and that within tribe Satyrini is ((((Lethina + Parargina) + Mycalesina) + Coenonymphina) +(Satyrina + (Melanargiina + Maniolina))). *L. deidamia*s together with *Pararge aegeria* are classfied into clade of subtribe Parargina (Figure 1). Interestingly, *Callerebia suroia* is classed into clade of Satyrini instead of Ypthimini. More mitogenome data of the two tribes will be helpful to make clear the real relationship.

**Figure 1:**
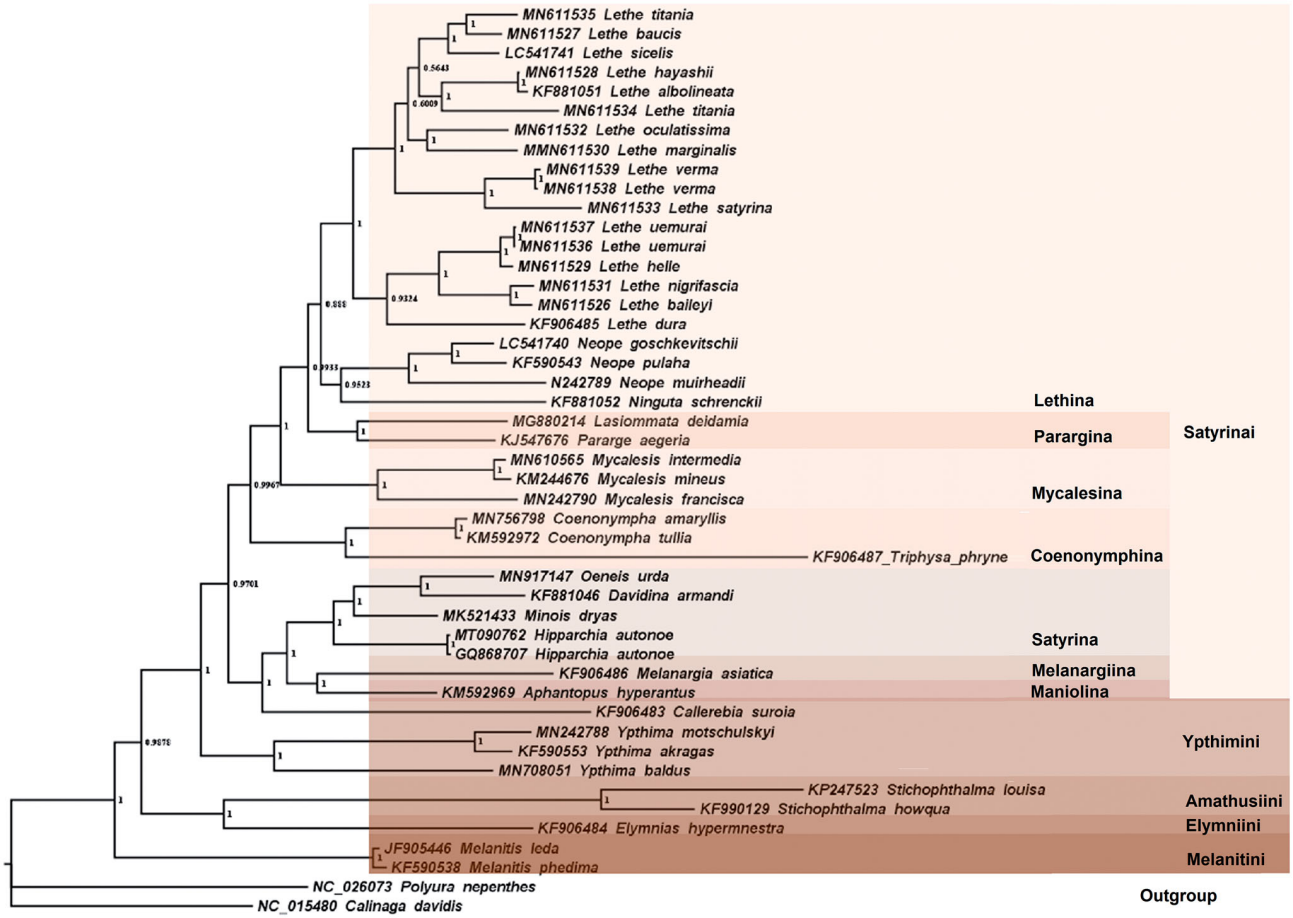
Phylogenetic tree for Satyrinae inferred with Bayesian Inference (BI) method.

## Depository

The specimen (accession number 20180630027) and the DNA solution (accession number 20180630027DNA) have been stored in the Specimens Room and the Human and Animal Genetics Laboratory college of Life Science, Huaibei Normal University, China. (Contact person Li Jun and email healthlicn@chnu.edu.cn)

## Supplementary Material

Supplemental Material

## Data Availability

The mitogenome sequence data that support the findings of this study are openly available in GenBank of NCBI at https://www.ncbi.nlm.nih.gov under the accession no. MG880214.
