# Supplementary material for: Complete mitochondrial genome of Lasiommata deidamia and its phylogenetic implication to subfamily Satyrinae (Lepidoptera: Nymphalidae)
Source: Mitochondrial DNA B Resour. 2021 Sep 15;6(10):2943–5. doi: 10.1080/23802359.2021.1955029 (PMC8451643; doi:10.1080/23802359.2021.1955029)
Supplement: Supplemental Material [file TMDN_A_1955029_SM7531.docx]

Supplementary table 1 List of primers.

| Primers | Primer sequences (5’-3’) | Recognition sites | Product length (bp) |
| --- | --- | --- | --- |
| F1 | AAACTAATAATCTTCAAAATTAT | 1281-1303 | 4803 |
| R1 | ACAGTGATATACCTCTAATTTG | 6062-6083 |  |
| F2 | TGCTCACCATTTGAATGTG | 5668-5686 | 2961 |
| R2 | TGCTTATTCTTCAGTCTGTCATAT | 8645-8668 |  |
| F3 | GAAGGAGGAGCTGCCATAT | 8371-8390 | 2302 |
| R3 | GCTCCATTAGCATGTAAAGTAC | 10651-10672 |  |
| F4 | TTGGATCATTATTAGCTTTATG | 10510-10531 | 4160 |
| R4 | CCAGCAGTTGCGGTTATACA | 14650-14669 |  |
| F5 | TCTAGAAACACTTTCCAGTACTTC | 14071-14094 | 2752 |
| R5 | GGGTTACCTAATTCTGTTCG | 1559-1578 |  |
